# Reversing pancreatic β-cell dedifferentiation in the treatment of type 2 diabetes

**DOI:** 10.1038/s12276-023-01043-8

**Published:** 2023-08-01

**Authors:** Jinsook Son, Domenico Accili

**Affiliations:** Department of Medicine and Naomi Berrie Diabetes Center, Vagelos College of Physicians and Surgeons, Columbia University, New York, NY 10032 USA

**Keywords:** Type 2 diabetes, Type 2 diabetes

## Abstract

The maintenance of glucose homeostasis is fundamental for survival and health. Diabetes develops when glucose homeostasis fails. Type 2 diabetes (T2D) is characterized by insulin resistance and pancreatic β-cell failure. The failure of β-cells to compensate for insulin resistance results in hyperglycemia, which in turn drives altered lipid metabolism and β-cell failure. Thus, insulin secretion by pancreatic β-cells is a primary component of glucose homeostasis. Impaired β-cell function and reduced β-cell mass are found in diabetes. Both features stem from a failure to maintain β-cell identity, which causes β-cells to dedifferentiate into nonfunctional endocrine progenitor-like cells or to trans-differentiate into other endocrine cell types. In this regard, one of the key issues in achieving disease modification is how to reestablish β-cell identity. In this review, we focus on the causes and implications of β-cell failure, as well as its potential reversibility as a T2D treatment.

## Diabetes: Regression of insulin-producing β-cells in the pancreas

Therapies aimed at preventing or reversing the regression of pancreatic insulin-producing cells could restore healthy sugar metabolism in individuals with type 2 diabetes. Jinsook Son and Domenico Accili from Columbia University, New York, USA, review how cellular stress and metabolic dysfunction can prompt β-cells in the pancreas to ‘dedifferentiate’, a process by which the specialized insulin-making cells transform into a more primitive state. The same triggers can also cause β-cells to ‘trans-differentiate’ into other cell types. In both cases, the pancreas loses β-cells, leading to compromised insulin production and impaired glucose metabolism. By understanding the underlying molecular mechanisms and signaling pathways involved in these processes, researchers hope to develop targeted therapies to inhibit or reverse the regression of pancreatic β-cells.

## Introduction

Diabetes is a chronic metabolic disease that poses a significant threat to public health, shortening life expectancy due to secondary complications such as cardiovascular disease, nephropathy, retinopathy, and neuropathy^1^. T2D accounts for nearly 90% of all diabetes cases and is marked by peripheral insulin resistance and subsequent pancreatic β-cell failure^2^. β-cells upregulate their insulin production and/or mass in response to increased metabolic demand under the insulin-resistant state^3^. However, persistent metabolic stress leads to a deterioration of β-cell function and mass^4^, which plays a significant role in the development of T2D^5^. β-cell dedifferentiation is a pivotal process underlying β-cell failure^6^, whereby β-cells undergo a loss of identity and dedifferentiate into nonfunctional endocrine progenitor-like cells^7–9^. Increasing evidence suggests that β-cell dedifferentiation is not an irreversible process^10–13^ and that dedifferentiated β-cells retain the capacity to redifferentiate into mature, functional β-cells^14,15^. This has also paved the way for the development of therapeutic strategies that aim to reverse β-cell dedifferentiation and promote β-cell regeneration as a means to treat T2D, including pharmacological agents that target specific molecular pathways involved in β-cell dysfunction and regeneration^15^. These promising strategies hold potential for improving T2D treatment by enhancing β-cell function and regeneration. In this review, we discuss the current understanding of the mechanisms underlying β-cell failure in T2D. We will explore the cellular consequences of β-cell failure, including β-cell dedifferentiation, and factors that contribute to this process. We will also review the evidence supporting the reversibility of β-cell dedifferentiation and the potential therapeutic strategies that target β-cell regeneration and function.

## Insulin resistance in the development of T2D

**Fig. 1:**
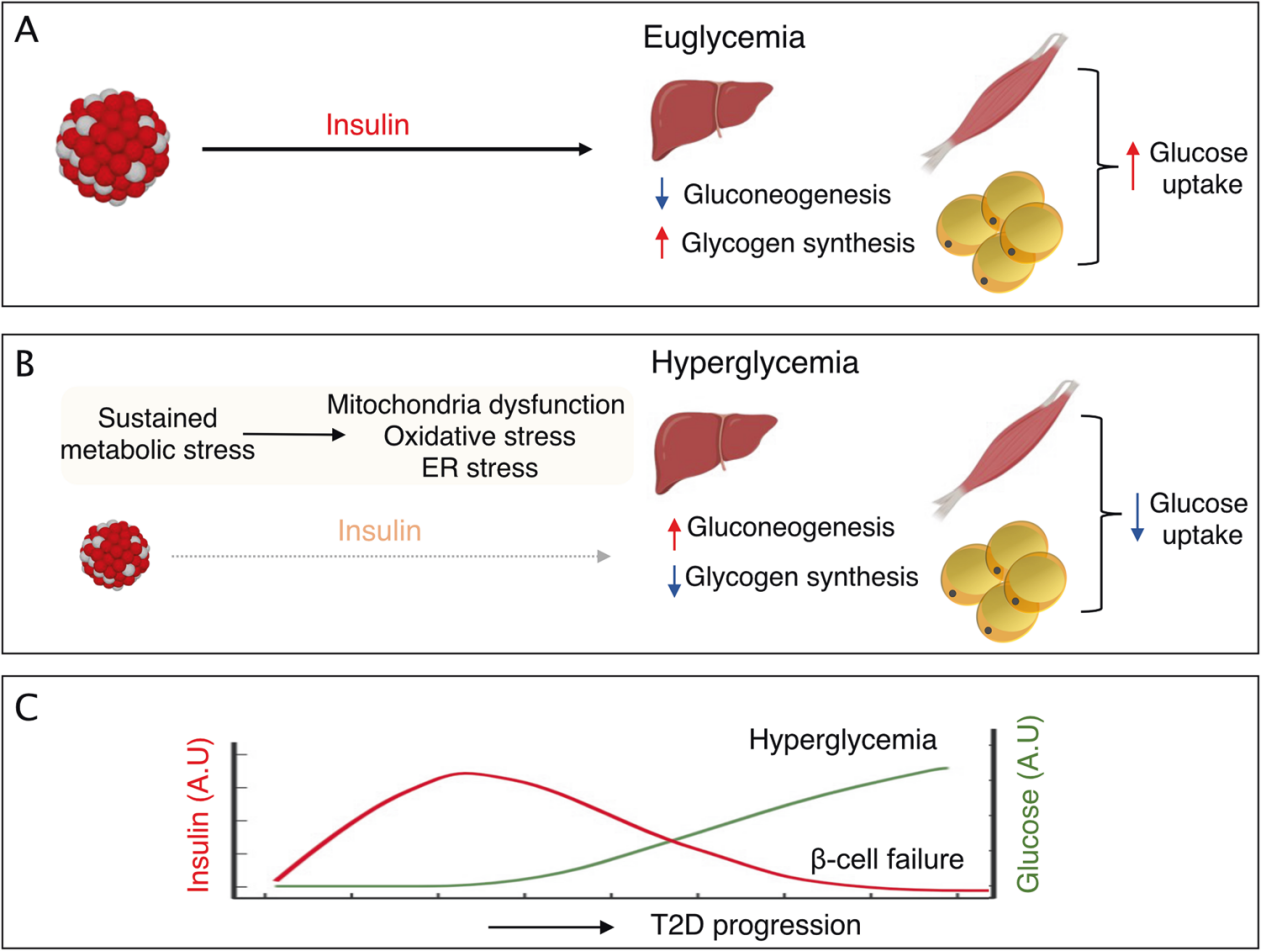
Progression of β-cell failure in T2D. **A** When blood glucose concentrations rise after a meal, β-cells secrete insulin, which activates peripheral tissues to take up glucose and maintain glucose homeostasis. For example, this process acts by reducing hepatic glucose output and gluconeogenesis (liver), inducing glycogen synthesis (liver and muscle), and increasing glucose uptake (muscle and adipocytes). **B**, **C** Metabolic stresses, such as excessive calorie intake, obesity, and physical inactivity, promote the development of insulin resistance, a condition in which peripheral tissues become less responsive to insulin. In response, β-cells produce and secrete an abnormally large amount of insulin (hyperinsulinemia). Sustained metabolic stress exacerbates ER and oxidative stress and mitochondrial dysfunction, which collectively contribute to β-cell failure and the loss of β-cell mass, leading to hyperglycemia.

When blood glucose concentrations rise after a meal, pancreatic islet β-cells secrete insulin, which activates peripheral glucose disposal and maintains glucose homeostasis^16^. For example, this process acts by reducing hepatic gluconeogenesis (liver), inducing glycogen synthesis (liver and muscle), and increasing glucose uptake (muscle and adipocytes) (Fig. 1)^17^. Insulin resistance is a pathophysiological condition characterized by impaired insulin action in insulin-sensitive target tissues^18^, wherein a normal insulin concentration fails to produce an effective insulin response^19^ (Fig. 1). Overnutrition, obesity, and physical inactivity are the major systemic causes of insulin resistance. At the molecular level, insulin resistance is caused by alterations in insulin signaling and its effectors^17^. Obesity, a common precursor to T2D, especially in North America and Western Europe, increases lipid accumulation and alters the secretion of adipokines, proinflammatory cytokines, and free fatty acids (FFAs)^20^. FFAs and proinflammatory cytokines act on metabolic tissues such as the liver and muscle, altering the inflammatory response and lipid profiles^21^. Proinflammatory cytokines can directly induce insulin resistance through different mechanisms, including a reduction in the number and catalytic activity of insulin receptors, an increase in Ser/Thr phosphorylation of insulin receptor and IRS, an increase in tyrosine phosphatase activity, primarily PTP-1B, which participates in receptor and IRS dephosphorylation, a decrease in PI3K and Akt kinase activity, and defects in GLUT-4 expression and function^20,22^. Insulin resistance in the liver results in impaired glycogen synthesis (glycogenesis), increased glucose production (glycogenolysis and gluconeogenesis), and excessive lipogenesis, which contribute to the development of fatty liver and its associated health risks^22^. In addition, insulin resistance decreases glucose uptake in muscle and fails to suppress lipolysis and FFA release in adipocytes, contributing to the increase in plasma FFA levels^21^.

To compensate for insulin resistance, pancreatic β-cells release more insulin, leading to hyperinsulinemia (Fig. 1). Paradoxically, hyperinsulinemia makes things worse by causing insulin receptor downregulation and activating a vicious cycle of gradually increasing compensatory insulin levels and decreased insulin signaling^2^. In fact, hyperinsulinemia is a hallmark of insulin resistance^23^. The inability of pancreatic β-cells to secrete enough insulin to overcome insulin resistance results in hyperglycemia^2^. The early stages of T2D are associated with hyperinsulinemia, but plasma insulin levels steadily drop as β-cell dysfunction progresses. This results in a relative lack of insulin and a reduction in β-cell mass in the advanced state of progression of the disease^24^ (Fig. 1).

## Causes of β-cell failure in T2D

Numerous hypotheses have been proposed to explain β-cell failure. Among these hypotheses, the most frequently invoked and experimentally validated are endoplasmic reticulum (ER) stress, mitochondrial dysfunction, and oxidative stress^25^.

### ER stress

Proinsulin is synthesized by β-cells using up to 50% of their translational capacity^26^. Increased β-cell insulin production intensifies the burden on the ER, where protein synthesis and folding take place. ER stress can be caused by the accumulation of misfolded proinsulin as a result of either excess proinsulin in the ER or ER alterations that decrease folding efficiency^27^. Cytokines and hyperlipidemia have been demonstrated to increase ER stress by causing ER Ca^2+^ depletion^28^, which leads to chaperone dysfunction and proinsulin misfolding^29^. To alleviate ER stress, cells activate the unfolded protein response (UPR), which increases the functional capacity of the ER by increasing enzymes and chaperones to facilitate protein folding while decreasing protein translation through activating eIF2 phosphorylation and mRNA degradation^30^. Notably, the UPR can exert both beneficial and detrimental effects on β-cell function, depending on the level of ER stress^27^. While mild to moderate ER stress can promote β-cell proliferation and insulin synthesis through UPR activation, sustained insulin demand due to insulin resistance can lead to excessive ER stress and terminal UPR activation^31,32^. This can result in β-cell dysfunction and apoptosis, contributing to the development of T2D. The mechanism responsible for the switch from beneficial to harmful UPR effects is not yet understood. However, it has been shown that chronic hyperglycemia promotes the overexpression of IRE1α, a central component of the UPR, which degrades proinsulin mRNA and contributes to β-cell failure^27^. Defective UPR can also contribute to the reduction of β-cell differentiation markers, such as Pdx1, NKX6.1, and Mafa, which can further impair insulin production^33^. A recent study showed that THADA (Thyroid Adenoma Associated), a T2D risk gene, can impair insulin secretion by reducing ER Ca2+ stores in the prediabetic phase^34,35^. Moreover, under sustained glucolipotoxicity-induced ER stress, THADA expression is upregulated and can activate a proapoptotic complex involving DR5, FADD, and caspase-8, ultimately exacerbating ER stress-induced apoptosis^35^.

### Oxidative stress

In β-cells, unlike other mammalian cells, glycolytic flow is tightly linked to elevated mitochondrial oxidative phosphorylation activity, where almost all glucose carbons are oxidized to CO_2_^36^. This is partly attributable to low levels of lactate dehydrogenase (LDH)^37^ and plasma membrane lactate/pyruvate transporter (MCT-1/Slc16a1)^38^ activity. Sustained glucose stimulation can augment glycolytic flux in β-cells, elevating reactive oxygen species (ROS) production^36^. Additionally, high FFA levels can increase ROS production in β-cells^39^. However, β-cells only express low levels of antioxidant enzymes, rendering them particularly vulnerable to ROS-induced damage^40^. Furthermore, hyperglycemia and/or hyperlipidemia lead to a reduction in antioxidant enzymes and a significant increase in superoxide, which ultimately results in the accumulation of ROS and oxidative stress in pancreatic β-cells^36^. Oxidative stress can lower insulin gene expression by impairing the DNA binding ability of PDX-1^41^ and affect the expression or activity of MafA, which is involved in β-cell function and maturation^42^.

### Mitochondrial dysfunction

β-cells take up glucose through the glucose transporter GLUT2 and carry out glycolysis via glucokinase to generate pyruvate. Pyruvate is transported into the mitochondria, where it produces ATP through the TCA cycle via oxidative phosphorylation^43^. As cytosolic ATP concentrations rise, ATP-sensitive K+ channels close, resulting in membrane depolarization and opening of voltage-dependent Ca2+ channels on the plasma membrane, allowing Ca2+ influx and insulin exocytosis^44^. Thus, mitochondria are both the primary sites of ROS generation and a critical component of glucose metabolism and insulin secretion in β-cells^44^. As a result, mitochondrial malfunction impairs GSIS and causes β-cell dysfunction^44^. Chronic hyperglycemia increases rates of glycolysis, the TCA cycle, and pyruvate oxidation^36^. This metabolic stress raises the mitochondrial membrane potential over a critical threshold, resulting in electron transport chain blockage at complex III and ROS generation^45^. Although mitochondrial mass may expand to compensate for metabolic stress, higher mitochondrial density has been shown to enhance ROS generation^46^. Mitophagy is a cellular process that clears damaged mitochondria^47^. Mitophagy-related genes have been shown to increase in prediabetic islets but decrease substantially in diabetic islets^48^. Failure to remove dysfunctional mitochondria due to impaired mitophagy can lead to oxidative stress. A recent genome-wide CRISPR screening has demonstrated that CALCOCO2, a T2D risk gene, plays a role in β-cell dysfunction^49^. CALCOCO2 promotes mitophagy activation, which helps β-cells tolerate metabolic stress^50^. Additionally, mutations in mitoribosomal proteins (MRPs) have been associated with diabetes, resulting in reduced oxidative phosphorylation capacity and ultimately leading to mitochondrial dysfunction^51^.

It is crucial to underline the tightly intertwined nature of these three factors and their potential to exacerbate one another, contributing to β-cell failure. Mitochondrial dysfunction is directly linked to oxidative stress and vice versa. The ER and mitochondria interact closely to form microdomains called mitochondria-associated membranes (MAMs)^52^. Miscommunication between these organelles can lead to mitochondrial dysfunction, ER stress, and altered calcium levels and ultimately affect β-cell function^52,53^.

## Cellular consequences of stressed and dysfunctional β-cells

**Fig. 2:**
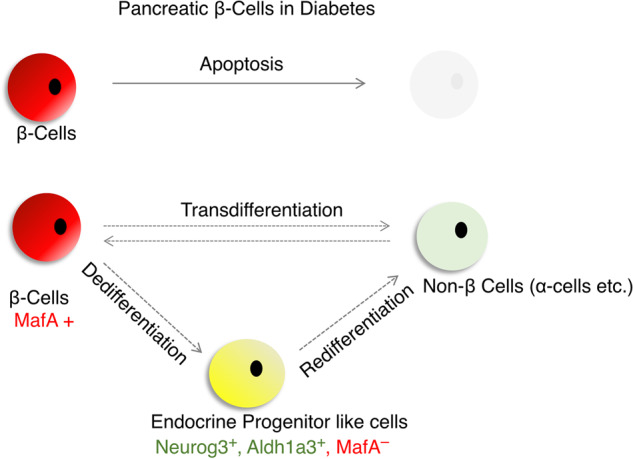
Dynamic changes in β-cell fate in the development of T2D. While apoptosis was previously thought to be the primary cause of β-cell mass loss in response to metabolic stress, recent evidence suggests that the fundamental cause is an alteration in β-cell identity due to dedifferentiation or transdifferentiation. These processes involve the loss of mature β-cell characteristics and the acquisition of alternative cell fates, leading to a reduced capacity for insulin secretion and impaired glucose metabolism.

Chronic metabolic stress leads to dysfunction of β-cells and consequent loss of β-cell mass (Fig. 1)^2^. The β-cell mass is 25–60% less in T2D patients than in weight-matched nondiabetic control individuals^54^. Conventionally and mechanically, β-cell death by apoptosis was thought to account for these features in response to metabolic stress^2^. Many studies have demonstrated that stressed β-cells are susceptible to apoptosis. However, rates of β-cell apoptosis are insufficient to explain the extent of β-cell loss observed in T2D^54^. Moreover, it has been shown that a few weeks of a low-calorie diet can partially restore β-cell function, even years after a T2D diagnosis, without affecting β-cell proliferation. This observation challenges the notion that β-cells are irreversibly lost and suggests that cell death alone cannot fully account for the loss of β-cells. As an alternative mechanism, experimental research and clinical data suggest that changes in β-cell fate could provide a plausible biological explanation for the decrease in β-cell mass (Fig. 2).

### β-cell dedifferentiation

Chronic hyperglycemia in pancreatectomized rats impairs β-cell differentiation, as indicated by reduced β-cell markers, such as Pdx1 and Nkx 6.1^55^. Lineage tracing of β-cells in diabetic animal models revealed that β-cells do not die but lose their ability to produce insulin^6,11,12,56–59^. They in fact revert to a progenitor-like cell via β-cell dedifferentiation, as evidenced by the reactivation of progenitor cell markers, such as Ngn3, Nanog, OCT4, and L-Myc, and the downregulation of β-cell differentiation markers^6,11,12,56–59^. In addition, lineage tracing studies have shown that some cells undergo transdifferentiation or redifferentiation to become α-like or mesenchymal cells (Fig. 2)^6,11,12,56–59^. Moreover, aldehyde dehydrogenase 1A3 (ALDH1A3) has been identified as a marker of β-cell dedifferentiation in β-cell-specific Foxo(s) knockout mice^60^, where its expression is activated in lineage-traced dedifferentiated β-cells. Thereafter, it was demonstrated that its expression is elevated in various diabetic mouse models with dedifferentiated β-cells, including LDB1_KO and Lepr mutant (db/db) mice^6,11,12,56–59^. Furthermore, when db/db mice are subjected to calorie restriction, ALDH1A3 expression is suppressed, whereas β-cell differentiation markers (Mafa, Foxo1 and NeuroD1) are upregulated^14^. These findings suggest that β-cell dedifferentiation is a protective mechanism against cell death in chronic hyperglycemia and that under certain circumstances, cells can redifferentiate into functioning β-cells^6,11,12,56–59^.

To determine the role of β-cell dedifferentiation in the pathophysiology of human T2D, it is essential to validate experimental animal models in human patients. Although the mouse is a useful genetic tool for studying diabetes, there are differences in the anatomy and physiology of human and rodent islets. Human islets have proportionally fewer β-cells and more α-cells than mouse islets, and β-cells are not clustered but intermingle with other endocrine islet cells^61^. Human islets also lack the typical synchronous Ca^2+^ oscillatory response observed in rodent islets^61^. Mouse and human islets differ significantly in their innervation patterns. In mouse islets, both sympathetic and parasympathetic axons innervate endocrine cells, whereas in human islets, sympathetic axons primarily contact smooth muscle cells of the vasculature^62^. It has also been shown that human islets are capable of accumulating lipid droplets (LDs), which are not found in mouse islets, even under hyperglycemic and hyperlipidemic conditions^63^. Considering these differences, it is crucial to investigate whether β-cell dedifferentiation occurs in human T2D to develop effective therapeutic interventions that can reverse this process and treat diabetes. Since in vivo lineage tracing is not possible in humans, it can be difficult to evaluate the plasticity of human endocrine pancreatic cells. However, it is still possible to survey the range of cell states that result in β-cell dedifferentiation. Consistent with this idea, reduced expression of Foxo1, Nkx6.1, and MafA has been shown in human T2D donors^9^. Ngn3 was not detected in these samples; however, consistent with animal research, the number of ALDH1A3-positive cells (dedifferentiated β-cells) is elevated in human T2D pancreases^9^. This observation has been confirmed by different clinical samples in European, North American^9^, Chinese^8^, and Japanese^7^ T2D populations^64^.

### β-cell transdifferentiation

Lineage tracing studies in mouse models further demonstrate that under hyperglycemic conditions, β-cells transdifferentiate into other islet cell types. In addition to the β-cell-specific FoxO1 inactivation in β-cells described above, lineage tracing of Nkx2.2 knockout β-cells in mice reveals that β-cells undergo transdifferentiation into α- or δ cells^65^. β-cells lacking Pdx1 lose β-cell differentiation markers but acquire α-cell-like characteristics by expressing glucagon and the α-cell-restricted transcription factor Arx^66^. Additionally, Arx activation in β-cells leads to transdifferentiation into α- or PP cells^67^. In cases of severe β-cell depletion, other islet cells can undergo transdifferentiation into β-cells^68^. For instance, lineage tracing experiments of α-cells in β-cell-ablated mice show conversion of α- to β-cells, suggesting that α-cells can sense β-cell mass^68^. Additionally, in mouse models, ectopic expression of PAX4^69^ or deletion of Arx^70,71^ in α-cells causes transdifferentiation into β-cells.

In addition to transdifferentiating between endocrine cells, exocrine cells have been shown to convert into β-cells. For example, pancreatic acinar cells overexpressing Ngn3, Pdx1, and Mafa simultaneously transdifferentiate into functional β-cells, which can reduce hyperglycemia in streptozotocin (STZ)-treated mice^72^.

The presence of multihormone-positive cells, such as INS and GCG, in pancreas sections of T2D patients supports the idea of islet cell transdifferentiation^9^. Moreover, human β-cells have been observed to spontaneously transform into α-cells or ductal cells during islet cell reaggregation or long-term in vitro culture^73,74^, but the relevance of these findings to T2D pathogenesis in humans remains unclear.

## Heterogeneity of islet cells in the wake of single-cell RNA sequencing studies

**Fig. 3:**
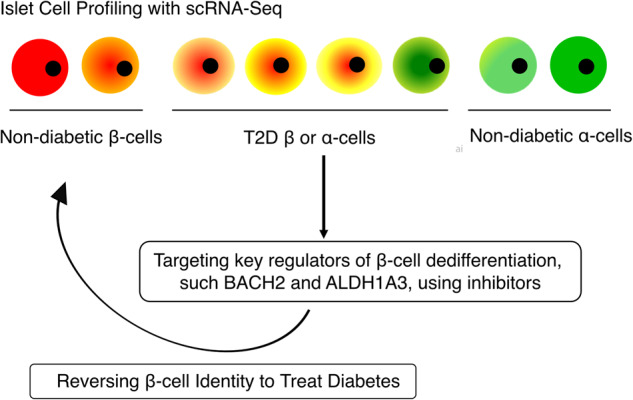
Reversibility of β-cell failure as a therapeutic approach. scRNA-Seq is a powerful tool for comprehensively understanding the repertoire of islet cells in both normal and T2D states. Identifying key master regulators of each cell type, including dedifferentiated β-cells, offers insight into potential therapeutic targets. Targeting key regulators of β-cell dedifferentiation, such as BACH2 and ALDH1A3, using small molecule inhibitors may hold promise as a potential strategy to reverse β-cell identity and treat T2D.

The occurrence of dedifferentiation and transdifferentiation in β-cells in response to chronic hyperglycemia suggests that islet cells have a high degree of plasticity. This offers the potential for developing therapeutic interventions that target the restoration of functional β-cells. To pave the way for the development of treatment strategies, it is necessary to understand the repertoire of islet cell heterogeneity that presents in T2D patients (Fig. 3). Single-cell RNA sequencing (scRNA-Seq) can be informative in this regard. Numerous scRNA-Seq studies have been performed to examine islet cell heterogeneity in nondiabetic or T2D islets in humans^75–78^. Segerstolpe et al. discovered five different β-cell subtypes defined by the expression of RBP4, FFAR4/GPR120, ID1, ID2, and ID3^75^. Although the five clusters are not exclusively associated with diabetes, the authors report that T2D β-cells exhibit notably lower levels of INS mRNA and reduced expression of FXYD2 (Na,K-ATPase gamma subunit), along with increased levels of GPD2, which is a crucial component of the NADH mitochondrial shuttle.^75^. Muraro et al. described a group of genes that identify distinct β-cell subtypes, including SRXN1, SQSTM1, and three ferritin subunits (FTH1P3, FTH1, and FTL), which are involved in the ER and oxidative stress responses^76^. Baron et al. reported evidence of β-cell heterogeneity resulting from differences in the expression of ER stress response genes, such as HERPUD1, HSPA5, and DDIT3^78^, as well as the β-cell differentiation markers UCN3 and MAFA. Additionally, Xin et al. identified distinct subpopulations of β-cells in islets from nondiabetic donors based on the combined expression patterns of UPR and INS^79^. The authors also discovered that β-cell maturation markers (ISL1, PDX1, MAFA, MAFB, NEUROD1, NKX2-2, and SIX3) and mitochondrial biogenesis genes (TFB2M) are differentially expressed in β-cell subgroups^79^. Moreover, scRNA-Seq studies have provided further evidence supporting the notion of β-cell dedifferentiation^80,81^. Wang and Avrahami show that the gene expression patterns of α- and β-cells in T2D islets are similar to those observed in juvenile donors, suggesting that cell dedifferentiation occurs during the progression of T2D^80,81^.

### Limitations of scRNA-Seq for capturing meaningful cell heterogeneity

To analyze the transcriptome at the single-cell level, transcripts from each cell were barcoded and then pooled together for sequencing. After sequencing the pooled transcripts, the total number of reads was divided by the number of cells, and each read was debarcoded to assign it to the specific cell from which it originated. As a result, the sequencing depth in scRNA-Seq is substantially lower than that in bulk RNA-Seq. The average number of genes detected in each cell ranges from a few hundred to a few thousands. Low-abundance RNAs will thus be detected by random chance in certain cells but not others. This phenomenon of gene “dropout” potentially results in the loss of biological information^82^, increased sampling errors and artifactual cell heterogeneity^83^. In fact, meta-analyses of five previous scRNA-Seq islet studies have revealed that only a small number of genes (<100) are consistently detected in all β-cells, while ~20 genes exhibit differential expression across β-cells. Additionally, the function of these differentially expressed genes has not been determined^84^. This work also highlights the fact that several key transcription factors, such as Mafa, are not consistently detectable in many β-cells, although the gene product of this mRNA is readily detected by immunohistochemistry in most β-cells^84,85^.

To overcome this problem, we applied a systems biology approach, ARACNe (Algorithm for the Reconstruction of Accurate Cellular Networks)^86^, to build protein activity analyses derived from scRNA-Seq (termed Single-cell Protein Activity Analyses) from human islets of normal and diabetic donors and metaVIPER (Virtual Inference of Protein-activity by Enriched Regulon analysis)^87,88^ to identify mater regulatory (MR) proteins controlling the formation of distinct cellular phenotypes that represent putative mechanistic determinants of aberrant, T2D-related transcriptional β-cell states^89^. MetaVIPER efficiently addresses the dropout issue since the activity of a protein is evaluated from >50 of its transcriptional targets, thereby allowing valid protein activity measurement even from low-depth profiles^87^. Thus, metaVIPER provides accurate, quantitative activity assessment of the function of proteins whose mRNA is not detected in a given cell, allowing the classification of key lineage markers in individual cells that would be missed at the gene expression level. Using this approach, we identified distinct cell types that reflect physiologic β- and α-cell states as well as aberrant cell states that are highly enriched in T2D patients. These aberrant states are characterized by metabolic inflexibility, a mixed β-/α-cell identity, and endocrine progenitor-like/stem cell features^89^ (Fig. 3). It is worth highlighting that our unsupervised analysis conducted in human T2D islets corroborated previous experimental animal data indicating β-cell dedifferentiation.

## Therapeutic strategies to treat T2D-associated β-cell failure

Treatment strategies for β-cell failure can be categorized into two groups: increasing cell number or enhancing insulin secretion. The former has been pursued by transplantation of cadaver islets^90^ or stem cell-derived β-cells^91^ in patients requiring immune suppression for unrelated organ transplant (primarily kidney). Stimulation of β-cell proliferation^92^ and inhibition of β-cell apoptosis have also been proposed, but there are no currently approved drugs to achieve this goal^8,93^. Drugs that promote insulin secretion have been used for decades but are plagued by secondary failures^24^.

The discovery of dedifferentiation as a feature of β-cell failure raised the question of whether the process is reversible and, if so, whether it represents an actionable treatment target^89^. Human studies in which low-calorie diets improve glucose homeostasis in T2D patients corroborate the notion that β-cell failure can be reversible for a long time even after disease onset^94–97^. In T2D patients, a low-calorie diet can restore glucose control, and chronic adherence to this diet has lasting benefits on glycemia^94–99^. Similarly, when diabetic *db/db* mice are compared to pair-fed wild-type littermates, glycemia levels decline and insulin secretion increases^14^. In addition, phlorizin treatment has been demonstrated to prevent hyperglycemia by restoring insulin mRNA expression and β-cell differentiation markers^55,100^.

These observations in humans and rodents are consistent with the possibility that β-cell function can be restored. Lineage tracing experiments with inducible Pdx1-cre or neurogenin3-cre also support this idea^10–13^. Our recent study using lineage tracing of ALDH1A3+ cells revealed that dedifferentiated β-cells can convert back to mature, functional β-cells^15^. Additionally, genetic and pharmacological ablation of ALDH1A3 in *db/db* mice has been shown to enhance β-cell function by increasing insulin secretion and β-cell proliferation (Fig. 3)^15^. Furthermore, CRISPR-mediated functional studies in human islets show that the T2D transcriptional signature can be reversed by targeted inhibition of a key master regulator of dedifferentiation, BACH2^89^, which we also characterized as an ectopically activated gene in FoxO1 knockout β-cells^60^. The administration of a BACH inhibitor reduced hyperglycemia in diabetic mice and restored β-cell function (Fig. 3)^89^. Because BACH inhibitors (which target both BACH2 and the related isoform BACH1) are FDA-approved for the treatment of disorders such as multiple sclerosis^101^, there is an immediate opportunity to investigate this pathway in human clinical trials^102^.

Thus, this proof-of-concept study opens a new avenue for understanding the reversal of disease progression and expands prospects for developing novel therapeutics for restoring β-cell function and identity in T2D.

## Conclusions and perspectives

Multiple factors, including oxidative and ER stress and mitochondrial dysfunction, contribute to the development of β-cell failure and altered β-cell identity. Thus, converting endocrine progenitor-like cells (dedifferentiated β-cells) to differentiated β-cells is an appealing therapeutic approach since it is reminiscent of the differentiation of β-cells that naturally takes place during development. Further research is needed to understand the similarities and differences between natural endocrine progenitors and dedifferentiated β-cells. Advances in technology and analytic tools for genome-wide studies at the single-cell level will assist in elucidating this point.

With a better understanding of the repertoire of islet cell heterogeneity in both normal and T2D subjects, we can identify disease-specific subpopulations and link them with genetic risk factors, paving the way for designing personalized precision-based treatments. In addition, a thorough investigation of islet cell subtypes in large human populations can help identify whether specific β-cell subpopulations are more susceptible to metabolic stress and failure as the disease progresses. This knowledge can lead to targeted therapies that aim to protect vulnerable populations from metabolic stressors and prevent the progression of the disease.
